# MASH-Associated T Cell Activation and Neuroinflammation: Evidence for a Hepatosystemic Immune Axis

**DOI:** 10.1055/a-2908-1230

**Published:** 2026-07-23

**Authors:** Britton S. Cartee, Fabiola Diniz, Ravi P. Rai, Pabitra B. Pal, Rahul Srivastava, Daniel Rossmiller, Reben Raeman, Smita S. Iyer

**Affiliations:** 1 Department of Pathology 6614 School of Medicine, University of PittsburghPittsburgh, PennsylvaniaUnited States; 2 Department of Pharmacology and Chemical Biology 6614 School of Medicine, University of PittsburghPittsburgh, PennsylvaniaUnited States; 3 Organ Pathobiology and Therapeutics Institute (OPTIn), School of Medicine, University of PittsburghPittsburgh, PennsylvaniaUnited States; 4 Pittsburgh Liver Research Center (PLRC), School of Medicine, University of PittsburghPittsburgh, PennsylvaniaUnited States

**Keywords:** T lymphocytes, liver–brain axis, cognitive decline, MASH, chronic metabolic diseases

## Abstract

“The liver is the origin of the veins and the source of the blood.”—De Usu Partium Galen.

Galen's hepatocentric physiology foreshadowed the liver–brain axis, a network linking vagal signaling, cytokines, metabolites, and migratory immune cells. Central to the immune arm of this axis, the liver generates 25 to 50% of thoracic duct lymph, positioning hepatic lymphatic output as a significant, though not yet fully characterized, source of T cells and cytokines in systemic circulation, a contribution that increases with hepatic inflammation in chronic metabolic diseases. We propose that T lymphocytes are the key cellular effectors through which inflammation originating in the liver, propagating systemically, a process we term hepatosystemic inflammation, reaches the central nervous system (CNS). In metabolic dysfunction-associated steatotic liver disease, hepatosystemic inflammation generates peripheral “push signals” that may license T cells for CNS access, while neuroinflammatory “pull signals” at CNS barrier interfaces may establish permissive entry points. In conclusion, we propose that T cell licensing by hepatosystemic signals represents the immune arm of the liver–brain axis and a mechanistic bridge between metabolic liver disease and neuroimmune dysfunction and associated cognitive decline, and identifies candidate therapeutic targets along this cascade.

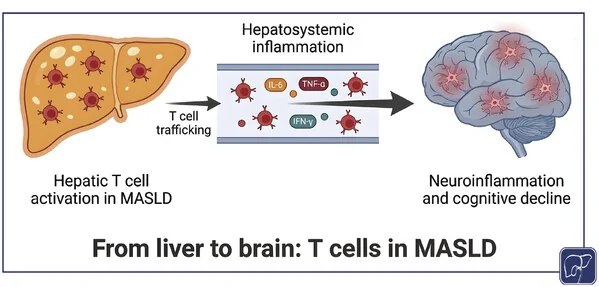

## Introduction

Hepatic encephalopathy established the liver–brain connection in clinical medicine, but its dependence on overt liver failure has long obscured a more fundamental relationship operating across the full spectrum of health and disease. 1 2 Metabolic dysfunction-associated steatotic liver disease (MASLD), previously designated nonalcoholic fatty liver disease (NAFLD), and diagnosed by hepatic steatosis in the presence of at least one cardiometabolic risk factor, offers a clearer window into this relationship. 3 4 Since MASLD and NAFLD designate the same individuals, the existing NAFLD literature applies to MASLD.

MASLD spans a pathological spectrum from simple hepatocyte triglyceride accumulation (steatosis) to metabolic dysfunction-associated steatohepatitis (MASH, formerly nonalcoholic steatohepatitis [NASH]), marked by active inflammation and fibrosis. 5 6 7 The disease affects approximately one-third of adults in Western populations, with prevalence reaching 31% in North America; between 20 and 33% of those affected progress to MASH. 8 Cirrhosis and hepatocellular carcinoma are downstream consequences, and MASH-related cirrhosis is now among the leading indications for liver transplantation in the United States. 9 10 Immune dysregulation is a driver of disease progression, yet the specific triggers of immune activation and the cellular mediators through which hepatic inflammation propagates to the central nervous system (CNS) are not well-defined. Given that MASLD is associated with cognitive impairment in nearly 50% of patients, at rates higher than those observed with obesity alone, 11 the mechanisms by which hepatic inflammation propagates through the systemic compartment, a process we term hepatosystemic inflammation, to reach the CNS demand investigation. This review traces the mechanistic path from hepatic immune activation to CNS pathology with T cells as the central link. (See Glossary)

The architecture of that path begins with the liver–brain axis. The liver and brain are connected at steady state through a signaling network regulated by neural circuits, soluble factors, and circulating immune cells (The Liver–Brain Axis section). Central to this interaction are T lymphocytes, which surveil the body through an expansive lymphatic network and, when activated, function as mobile effectors bridging hepatic inflammation and neuroimmune responses (T Cell Biology: Education, Differentiation, and CNS Trafficking **section** ). The clinical relevance of this axis is illustrated by epidemiological evidence linking MASLD to cognitive decline (Epidemiological Evidence Linking MASLD to Cognitive Decline **section** ), findings corroborated by postmortem studies (Postmortem Studies of CNS T Cell Influx in MASLD **section** ), and mechanistic experimental studies in animal models (Hepatosystemic Inflammation Drives Neuroinflammation in MASLD: Evidence from Animal Studies **section** ). These data position T cell-mediated liver–brain interactions as an underappreciated mechanism in the neurological sequelae of chronic metabolic disease, with implications for therapeutic strategies targeting neurodegeneration (Therapeutic Implications and Future Directions **section** ).

### The Liver–Brain Axis

#### Neural Pathways

The liver–brain axis operates through overlapping neural, humoral, bile acid, and metabolite signaling pathways ( Fig. 1 ). These regulate glucose metabolism, energy homeostasis, feeding behavior, and neurological function, particularly through hypothalamic circuits that integrate nutrient sensing with appetite regulation. 12 13 The vagus nerve ( Fig. 1A ) is the principal circuit for bidirectional communication between the liver and the brain, 14 15 16 functioning as the hepatoportal nutrient-sensing system, a specialized interface that detects nutrient flux and conveys this information to the CNS before nutrients reach systemic circulation. 16 Afferent vagal fibers innervating the hepatic portal vein sense portal glucose, 17 18 free fatty acids, 19 and amino acids 20 from the portal vein adventitia, and intra- and extrahepatic bile ducts. 21 22 Glucose sensing depends on glucose transporter 2-mediated uptake and glucokinase activity, which together alter intracellular adenosine triphosphate (ATP) levels and modulate ATP-sensitive potassium channels in vagal sensory terminals. 23 24 25 26

**Fig. 1:**
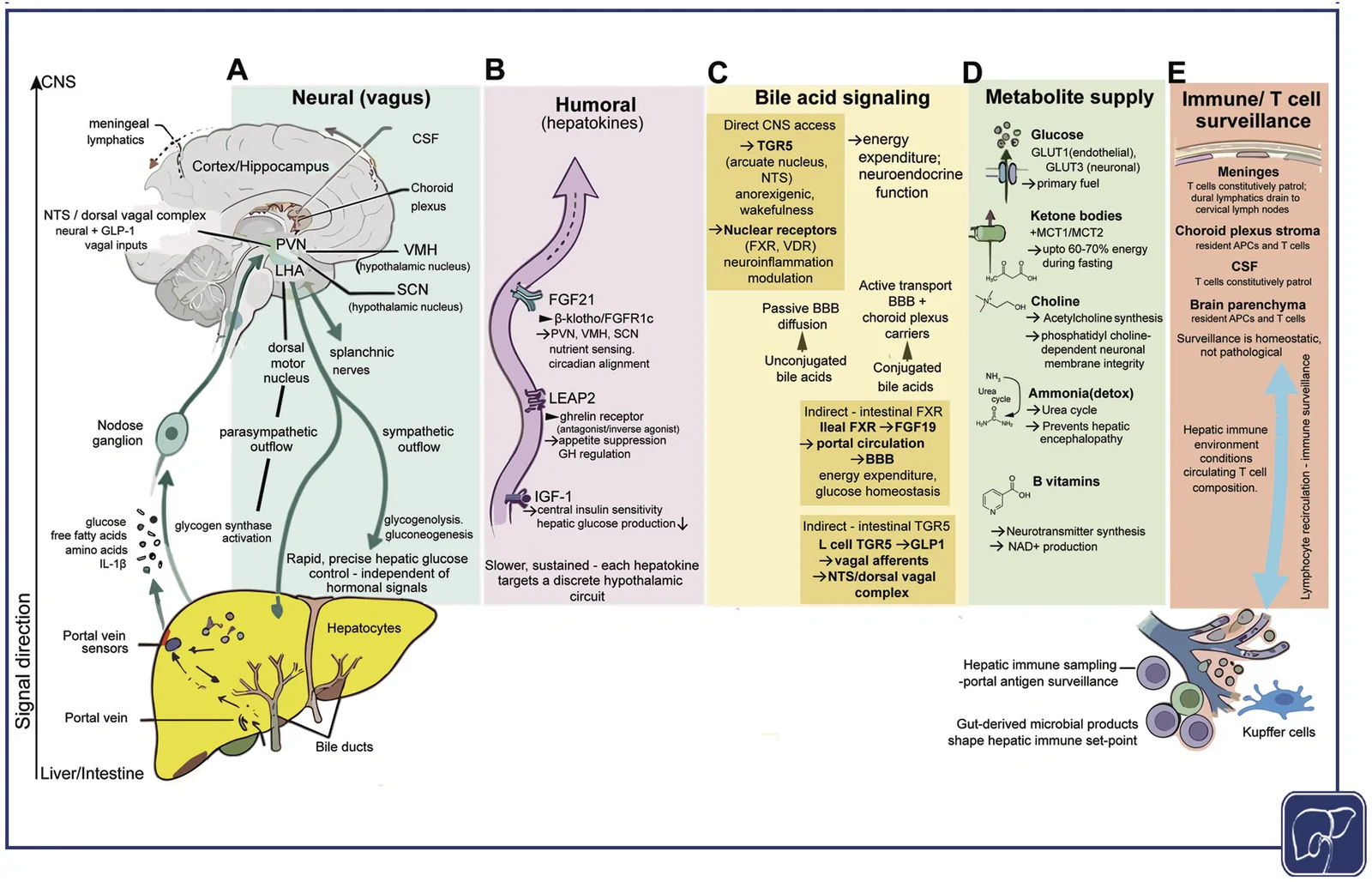
The liver–brain axis. The liver communicates with the brain through five parallel signaling routes: ( **A** ) vagal neural circuits, ( **B** ) hepatokine-mediated humoral signaling, ( **C** ) bile acid signaling, ( **D** ) metabolite supply, and ( **E** ) immune/T cell surveillance. Each route targets discrete CNS circuits to regulate glucose homeostasis, energy balance, feeding behavior, and neurological function. Under physiological conditions, T cell recirculation between the systemic compartment and CNS border compartments is homeostatic and nonpathological. In metabolic disease, this immune axis becomes dysregulated and drives neuroinflammation ( Figs. 2 3 4 ). APC, antigen-presenting cell; BBB, blood–brain barrier; CSF, cerebrospinal fluid; FGF19/21, fibroblast growth factor 19/21; FXR, farnesoid X receptor; VDR, Vitamin D receptor; GH, growth hormone; GLP-1, glucagon-like peptide-1; GLUT1/3, glucose transporter 1 and 3; IGF-1, insulin-like growth factor 1; LEAP2, liver-expressed antimicrobial peptide 2; LHA, lateral hypothalamic area; MCT1/2, monocarboxylate transporter 1/2; NTS, nucleus of the solitary tract; PVN, paraventricular nucleus; SCN, suprachiasmatic nucleus; TGR5, Takeda G protein-coupled receptor 5; VMH, ventromedial hypothalamic nucleus.

These signals travel through the nodose ganglion 27 to the dorsal vagal complex in the brain stem, including the nucleus of the solitary tract (NTS) and dorsal motor nucleus of the vagus. 28 29 30 The NTS, a hub for visceral sensory input, receives and coordinates hepatic signals with gastrointestinal and hormonal inputs and projects to hypothalamic nuclei such as the arcuate nucleus and paraventricular nucleus (PVN). 31 32 33 Through these projections, hepatic signals influence neuropeptide systems including proopiomelanocortin (POMC) and neuropeptide Y/agouti-related peptide (NPY/AgRP), which regulate feeding behavior and energy expenditure. 34 Hepatic vagal inputs activated by portal glucose or lipid signals preferentially engage anorexigenic POMC neurons while suppressing orexigenic NPY/AgRP activity, reducing food intake. 35 36 More recently, hepatic vagal afferents are also implicated in the regulation of circadian feeding behavior, conveying clock-dependent signals that modulate feeding patterns across the light–dark cycle. 37 Thus, signals from the dorsal vagal complex are integrated with hormonal, motivational, and circadian inputs. 38 Descending vagal efferents close the loop: parasympathetic vagal outflow, driven primarily by the lateral hypothalamic area via the dorsal motor nucleus, promotes hepatic glycogen synthesis by activating glycogen synthase, 39 in part through modulation of insulin signaling and enhanced hepatic glucose uptake. In contrast, sympathetic noradrenergic outflow, driven primarily by the ventromedial hypothalamic (VMH) nucleus via splanchnic nerves, promotes hepatic glucose output through glycogenolysis 40 and gluconeogenesis. 41 The PVN contributes to both sympathetic and parasympathetic projections to the liver. 38 Together, this neural architecture enables rapid and precise hypothalamic control over hepatic glucose flux, operating independently of slower hormonal signals, permitting dynamic adaptation of hepatic metabolism to changing nutritional and physiological states. Beyond vagal nutrient-sensing circuits, the amygdala contributes a stress-responsive neural arm of the liver–brain axis. 42 Acute stress activates medial amygdala neurons projecting to the VMH, which drive hepatic gluconeogenesis through a polysynaptic connection to the liver via the sympathetic nervous system, independent of adrenal or pancreatic glucoregulatory hormones. Repeated stress disrupts this circuit, producing diabetes-like dysregulation of glucose homeostasis. In addition to nutrients, hepatic vagal afferents relay inflammatory signals, specifically the pyretic cytokine interleukin-1β (IL-1β) in a dose-dependent manner. 43

#### Humoral Pathways

Circulating hormones provide a slower but sustained layer of liver–brain communication. Hepatokines (cytokines, proteins, and hormones produced by the liver) engage discrete central circuits in accordance with their distinct functions: energy sensing, appetite regulation, and growth-coupled insulin sensitivity ( Fig. 1B ). Fibroblast growth factor 21 (FGF21), induced by dietary sugars and protein restriction, acts as a metabolic sensor via β-klotho/FGFR1c receptor complex expressed in the PVN, VMH, and suprachiasmatic nucleus (SCN), linking nutrient availability to both neuroendocrine functions and circadian alignment. 44 45 Liver-expressed antimicrobial peptide 2 (LEAP2), in contrast, operates primarily at the level of appetite regulation. Functioning as an endogenous competitive antagonist and inverse agonist of the ghrelin receptor, LEAP2 suppresses ghrelin-induced food intake and growth hormone secretion, with circulating levels rising postprandially and in obesity, and falling with fasting. 46 Insulin-like growth factor 1 (IGF-1), the principal growth hormone-regulated hepatokine, bridges growth signaling and insulin sensitivity: intracerebroventricular IGF-1 lowers hepatic glucose production and improves central insulin sensitivity. 47 Glycosylphosphatidylinositol-specific phospholipase D1 (GPLD1), a liver-derived exerkine induced by exercise, acts on brain endothelial cells by cleaving tissue-nonspecific alkaline phosphatase (TNAP) from their luminal surface. 48 Age-related accumulation of cerebrovascular TNAP impairs blood–brain barrier (BBB) transport and cognition; increasing GPLD1 or inhibiting TNAP restores barrier integrity and reverses aging- and Alzheimer's-related cognitive deficits in preclinical models. Whether MASLD-associated reductions in physical activity and hepatic function shift the balance from this protective axis toward pathogenic hepatosystemic signaling is an open question.

#### Bile Acid Pathways

Bile acids, synthesized from cholesterol by hepatocytes, also function as signaling molecules ( Fig. 1C ) that reach the brain through both direct and indirect routes. 49 Unconjugated bile acids may diffuse passively across the BBB, with cerebral levels correlating with serum concentrations, while conjugated bile acids require active transport via carriers expressed at the BBB and choroid plexus. 50 Within the brain parenchyma, bile acids engage the nuclear receptor Farnesoid X receptor (FXR) and the membrane receptor Takeda G protein-coupled receptor 5 (TGR5), activating energy expenditure and neuroendocrine pathways. 51 52 Bile acids also signal indirectly through intestinal pathways: intestinal FXR activation induces FGF19, while intestinal TGR5 stimulates glucagon-like peptide-1 (GLP-1) secretion from enteroendocrine L-cells; both mediators signal to the CNS via circulation and vagal afferents. Under physiological conditions, these indirect pathways, particularly TGR5 to GLP-1, represent the predominant mode of bile acid–brain communication.

#### Metabolite Pathways

The liver sustains brain function through the regulated production and export of key metabolites 13 ( Fig. 1D ). Glucose, maintained by hepatic gluconeogenesis and glycogenolysis, is the primary fuel for the brain, as neurons largely lack the capacity for β-oxidation of fatty acids for energy. 53 Glucose crosses the BBB via GLUT1 on endothelial cells and GLUT3 on neurons. 54 During fasting or carbohydrate restriction, the liver switches to producing ketone bodies, β-hydroxybutyrate and acetoacetate, which cross the BBB via monocarboxylate transporters (MCT1/MCT2), satisfying up to 60 to 70% of cerebral energy demands during prolonged starvation. 55 56 The liver also supplies choline, a precursor for both acetylcholine and phosphatidylcholine synthesis, of which the latter is incorporated into neuronal membranes. Consequently, choline deficiency impairs synaptic function and cognition. 57 58 Additionally, the liver maintains the neurochemical environment by detoxifying ammonia through the urea cycle, preventing the hyperammonemia that drives hepatic encephalopathy, and processing B vitamins essential for neurotransmitter synthesis and NAD ^+^ production. 13

The liver–brain axis comprises interconnected regulatory modules that converge on shared hypothalamic circuits to generate a unified metabolic response. Dysregulation at any node carries pathological consequences; vagal neuropathy disrupts afferent nutrient sensing and efferent hepatic control; altered bile acid metabolism perturbs central FXR/TGR5 signaling; hepatic dysfunction compromises glucose, ketone, and choline supply on which the brain depends. These disruptions underlie the metabolic inflexibility and neurological vulnerability observed in obesity, type 2 diabetes, MASLD, and hepatic encephalopathy, consistent with the centrality of the liver–brain axis to the integrative physiology of metabolic disease. 59 Hepatic inflammation and the immune remodeling that results are less-examined drivers of this dysregulation. T cell biology ( Fig. 1E ) therefore provides the mechanistic framework through which hepatic inflammation translates to cognitive decline in MASLD. While this review centers on T cells as the cellular effectors of hepatosystemic-to-CNS propagation, they operate within a broader inflammatory network: Kupffer cells (KC) and recruited monocytes generate the proximal hepatic cytokine signal, neutrophils amplify early hepatic injury, natural killer/natural killer T cells shape the intrahepatic cytokine milieu, B cells contribute through antibody-dependent and antigen-presenting functions, and microglia serve as the CNS sensor and amplifier of peripheral inflammatory input.

### T Cell Biology: Education, Differentiation, and CNS Trafficking

Immune cells constitute a growing component of the liver–brain axis, carrying metabolic and inflammatory signals across the liver and circulation as they access the CNS. As long-lived cells, spanning multiple differentiation and helper states, each with distinct functional profiles, T cells function as durable mediators of hepatosystemic-to-CNS inflammatory propagation. Where “The Liver–Brain Axis section” established the anatomical and physiological routes through which the liver communicates with the brain, T cells may exploit and modify these same routes during MASLD, functioning as a disease-amplifying immune module that converts hepatic inflammation into CNS pathology.

#### Education in the Thymus

T cell progenitors arise in the fetal liver and adult bone marrow, seeding the thymus throughout life, where they undergo random rearrangement of T cell receptor (TCR) genes to encode a T cell repertoire with enormous diversity. 60 61 To ensure a self-tolerant, major histocompatibility complex (MHC) or human leukocyte antigen (HLA)-restricted repertoire, thymocytes are selected based on the binding strength of their TCR to self-peptide-MHC complexes. 62 Cells whose TCRs fail to engage self-MHC sufficiently do not receive survival signals and die by neglect. TCRs with weak-to-intermediate binding for MHC-II or -I and self-peptide are positively selected and commit to either the CD4 or CD8 lineage, respectively. Among positively selected thymocytes, those bearing high-affinity TCRs for self-antigens are eliminated through negative selection. 63 A subset of self-reactive CD4 thymocytes receiving strong TCR signals is diverted to a Forkhead box P3(FOXP3 + ) regulatory T cell (Treg) fate, generating a subset dedicated to immune regulation. 64 As described in “Hepatosystemic Inflammation Drives Neuroinflammation in MASLD: Evidence from Animal Studies section,” Treg loss in MASLD is associated with immune activation in the liver and within the CNS.

The T cell selection process sculpts a mature, naive T cell repertoire that is self-tolerant and functionally competent. Upon thymic egress, naive T cells populate secondary lymphoid tissues, the spleen and lymph nodes, where immune responses are initiated. Because each TCR recognizes a specific peptide-MHC combination, the frequency of naive T cells specific for any given epitope is extremely low: approximately 1 in 10 ^5^ to 10 ^6^ naive T cells, corresponding to tens to a few hundred antigen-specific cells per mouse, 65 and roughly 0.2 to 10 per million naive T cells in humans, depending on the epitope and HLA allele. 66 Therefore, immune defense depends on robust clonal expansion and effector differentiation following antigen encounter, a process that also imprints the capacity of effector T cells to enter nonlymphoid tissues for immunosurveillance. 67

#### Activation in Secondary Lymphoid Tissues

Following thymic egress, naive T cells enter the recirculating pool, trafficking continuously between blood, lymph nodes, and spleen in search of cognate antigen, completing one to three circuits per day with typical lymph node dwell times of 12 to 21 hours, shorter for CD4 than CD8 T cells. 68 69 70 When a naive T cell encounters its cognate antigen (TCR recognition of peptide presented in the context of MHC) and receives co-stimulatory signals, an activation program is initiated, driving clonal expansion and effector differentiation. 71 T cell activation integrates three coordinated signals: signal 1, TCR recognition of peptide–MHC complex; signal 2, CD28 ligation by B7–1 (CD80) and B7–2 (CD86); and signal 3, polarizing cytokines. 72 These inputs upregulate the high-affinity IL-2 receptor α-chain (CD25), supporting autocrine IL-2 signaling that sustains proliferation, acquisition of effector programs (cytokine production and cytolysis), and induction of tissue-specific homing receptors that permit egress to nonlymphoid sites.

Several surface and nuclear markers define this activated state and are used extensively to identify activated T cells in experimental settings. K _i_ -67, a nuclear protein expressed during cell division, marks proliferating T cells. CD69, one of the earliest surface markers induced following TCR engagement, transiently retains activated T cells within the lymph node by interfering with sphingosine-1-phosphate receptor 1 (S1PR1)-mediated egress. CD69 physically associates with S1PR1 and promotes its internalization, thereby overriding the egress signal until T cell activation is sufficiently advanced. 73 CD69 expression on T cells in nonlymphoid tissues is frequently associated with tissue residence for precisely the same reasons, but CD69 alone is insufficient to infer a tissue-resident phenotype. CD44 is also an activation marker, but in contrast to CD25 and CD69, it is durably upregulated following antigen encounter in mice and remains elevated after antigen clearance, distinguishing antigen-experienced from naive T cells. These markers are invoked in subsequent sections to characterize hepatic and CNS T cell populations in MASLD.

In CD4 T cells, the cytokine milieu during priming dictates differentiation into functionally distinct helper subsets: predominantly T _h_ 1, T _h_ 2, T _h_ 17, or induced-Treg, a fate decision that impacts the ensuing immune response. 74 In addition to cytokine polarization, the identity of the antigen-presenting cell (APC), together with the anatomical origin of the draining lymph node, impacts the induction of trafficking receptors during differentiation, thereby determining which tissues the resulting effector T cells are licensed to enter. 75

#### Effector T Cell Migration: Surveilling Nonlymphoid Tissues

Having acquired effector functions and tissue-homing receptor programs during priming, activated T cells must exit secondary lymphoid organs and navigate to their target tissues. This egress is initiated in part by the downregulation of chemokine receptors such as C–C chemokine receptor (CCR7), allowing activated T cells to return to circulation via the efferent lymphatics and thoracic duct. 76 The concurrent upregulation of selectin ligands, integrins, and chemokine receptors, whose specific combination is imprinted during priming, determines which tissues will accept the circulating effector cell.

Not all tissues are equally accessible; however, nonlymphoid sites are classified into three functional categories based on their accessibility to effector and memory T cells. 77 78 Permissive tissues, such as the liver, are readily accessible to both effector and memory T cells without requiring local inflammation or antigen recognition; the sinusoidal architecture of the hepatic vasculature permits T cell influx independent of conventional homing programs. Effector-permissive tissues, as their designation implies, are accessible to activated/effector T cells during the active effector phase, when homing receptors are expressed, but circulating resting memory T cells have limited access at steady-state. The perivascular and border spaces of the CNS fall into this category, remaining permissive to activated T cells during an ongoing immune response, independent of antigen specificity. During acute SARS-CoV-2 infection, despite the lack of antigen, K _i_ -67 ^+^ activated T cells infiltrate the cerebrospinal fluid (CSF). 79 Restrictive tissues, by contrast, such as the skin and urogenital tract, lack tissue-tropic chemokines or adhesion molecules at steady-state and are accessible only when local inflammation induces the expression of chemokines such as (C–X–C motif chemokine ligand 9) CXCL9, CXCL10, and CCR5 ligands, largely through the actions of type I interferons (IFN) and IFNγ.

#### T Cell Migration to the CNS

Across tissue types, T cell extravasation follows a conserved multistep adhesion cascade. Initial contact is established by selectin-mediated tethering and rolling, in which P-selectin glycoprotein ligand-1 (PSGL-1) on T cells engages P-selectin and E-selectin on activated endothelium, decelerating circulating T cells. Genetic deletion of PSGL-1 or of E- and P-selectin does not prevent CNS T cell entry during neuroinflammation, indicating that rolling, while fostering initial contact, is dispensable during neuroinflammation. 80 81 82 Firm arrest follows upon chemokine-triggered inside-out integrin activation, mediated by G-protein-coupled receptor signaling through Rap1, which converts integrins to high-affinity conformations. 83 Key chemokine-receptor pairs driving this step include CXCL12–CXCR4, CCL2–CCR2, CCL5–CCR1/CCR5, and CXCL9/10/11–CXCR3. Firm adhesion is then established by α _4_ β _1_ integrin (or very late antigen 4, VLA-4) binding to vascular cell adhesion molecule-1 (VCAM-1) on the BBB endothelium. Natalizumab, which blocks this interaction, is FDA-approved for multiple sclerosis. 84 However, in simian immunodeficiency virus (SIV) infection in macaques, α4 blockade impairs activated CD8 T cell but not CD4 T cell migration to the brain parenchyma, suggesting context-dependent reliance on this pathway for parenchymal T cell entry. 85 Following firm arrest, leukocyte function-associated antigen (LFA-1 or α _L_ β _2_ ) engages intercellular adhesion molecule-1 (ICAM-1) and ICAM-2 on the BBB to mediate crawling against the direction of blood flow until the T cell identifies a permissive site for diapedesis, after which transendothelial migration proceeds either paracellularly or through transient transcellular pores that form around the migrating leukocyte without requiring gross barrier disruption. 86

#### CNS Barriers

The mechanics of extravasation described above operate within a layered CNS barrier, which is conceptually likened to a medieval castle with two concentric defensive walls. 87 The BBB and blood–cerebrospinal fluid barrier (BCSFB) constitute the outer wall, formed by tight junction-linked endothelial and epithelial cells, respectively ( Fig. 2A ). The inner wall is the glia limitans, composed of astrocytic end feet overlying a parenchymal basement membrane. Between these two walls lies the moat, the perivascular and subarachnoid spaces drained by CSF, where immune cells patrol during homeostasis without disturbing the adjacent parenchyma. T cells constitutively survey the meninges, subarachnoid space, and choroid plexus, and the dural meninges contain fenestrated blood vessels and conventional lymphatics draining to deep cervical lymph nodes, supporting organized afferent and efferent immune connections with the periphery. 88 89 90 91

**Fig. 2:**
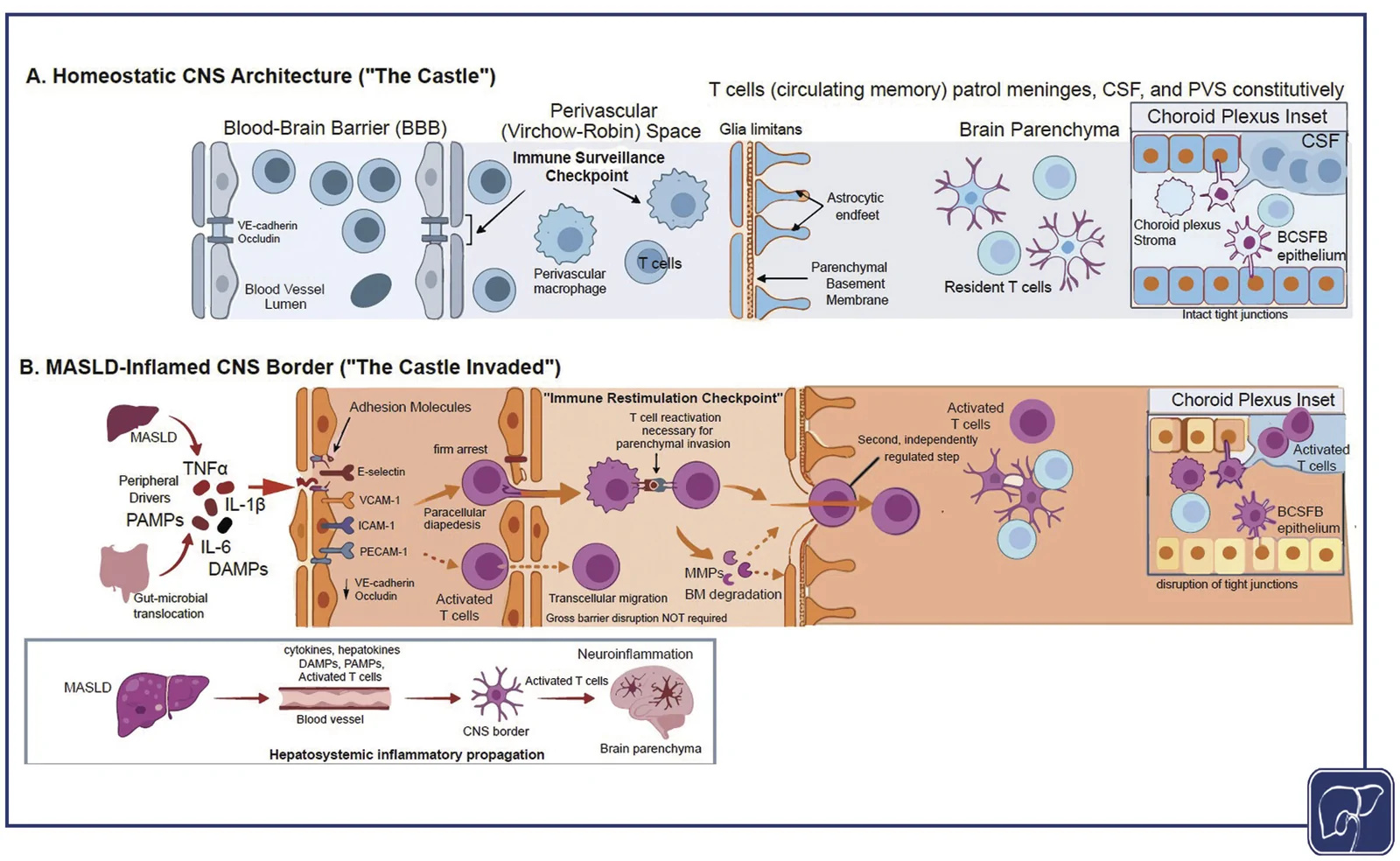
CNS border compartment remodeling in MASLD. Under homeostatic conditions ( **A** ), circulating T cells patrol the meninges, CSF, and perivascular space but are excluded from the parenchyma. The perivascular (Virchow–Robin) space and glia limitans constitute sequential checkpoints for parenchymal access. In MASLD ( **B** ), systemic release of TNF-α, IL-1β, IL-6, DAMPs, PAMPs, and gut-derived microbial products acts on BBB endothelium to downregulate occludin and VE-cadherin and upregulate E-selectin, VCAM-1, ICAM-1, and platelet endothelial cell adhesion molecule-1, lowering the threshold for T cell arrest and diapedesis. Activated T cells cross the endothelium via paracellular and transcellular routes, require APC-mediated restimulation in the perivascular space before parenchymal entry, and breach the glia limitans through MMP-dependent basement membrane degradation. At the choroid plexus, TNF-α and IL-1β disrupt epithelial tight junctions, providing a parallel BBB-independent entry route into the CSF compartment. BBB, blood–brain barrier; BCSFB, blood–CSF barrier; CSF, cerebrospinal fluid; DAMP, damage-associated molecular pattern; PAMP, pathogen-associated molecular pattern; APC, antigen-presenting cell; MMP, matrix metalloproteinase; PVS, perivascular space.

At the BCSFB, resident macrophages and dendritic cells in the choroid plexus function as sentinels, sampling the CSF and presenting antigen to T cells. The perivascular Virchow-Robin spaces represent a critical regulatory checkpoint between the endothelial BBB and the glia limitans, where perivascular APCs can restimulate T cells locally, and this reactivation is a prerequisite for parenchymal invasion. 92 93 Entry into the parenchyma proper therefore requires a second, independently regulated step across the glia limitans, supported by matrix metalloproteinase-mediated degradation of basement membrane components. 94 95

Systemic inflammation accelerates and amplifies T cell entry into the CNS through multiple convergent mechanisms acting on this architecture. Systemic inflammation following low-dose lipopolysaccharide challenge in mice induces key CNS-homing integrins VLA-4 and LFA-1 in circulating T cells, resulting in the influx of activated K _i_ -67 ^+^ T cells to the brain parenchyma. 85 Proinflammatory cytokines, particularly tumor necrosis factor-α (TNF-α), IL-1β, and IFNs, compromise BBB tight junction integrity by reducing occludin and VE-cadherin expression, increasing paracellular permeability. 96 97 Simultaneously, these cytokines drive upregulation of adhesion molecules on the endothelium, including E-selectin, VCAM-1, ICAM-1, and platelet endothelial cell adhesion molecule-1, lowering the threshold for T cell firm arrest and diapedesis. 98 99 The responsiveness of CNS borders to systemic inflammation is of importance to metabolic diseases, where chronic systemic inflammation provides precisely the signals required to license CNS immune cell entry.

Working model: in the context of MASLD, hepatic inflammation generates the systemic push signals: activated, α _4_ β _1_ -integrin-competent T cells primed for CNS influx, while neuroinflammation provides the pull signals, upregulating endothelial adhesion molecules such as VCAM-1 and chemokine gradients at border compartments. MASLD generates a chronic low-grade systemic inflammatory state characterized by the same signals that upregulate endothelial adhesion molecules, compromise tight junction integrity, and license peripheral immune cell entry into CNS border compartments ( Fig. 2B ). Unlike acute liver failure or end-stage cirrhosis, where ammonia toxicity and overt hepatic encephalopathy dominate the neurological picture, MASLD represents a far more prevalent and insidious exposure, one in which low-level but persistent systemic inflammation may exert cumulative effects on the CNS immune landscape over decades. This prediction is testable: patients with MASLD should show elevated rates of cognitive decline relative to metabolically healthy individuals, and these associations should track with disease severity and inflammatory burden.

### MASLD Is Linked to Neuroinflammation and Cognitive Decline: Evidence from Human Studies

#### Epidemiological Evidence Linking MASLD to Cognitive Decline

MASLD is associated with deficits across multiple cognitive domains: memory, processing speed, attention, and executive function prior to overt hepatic decompensation, a pattern consistent with systemic inflammatory disease rather than end-stage liver failure. 100 101 102 103 MASLD severity assessed by Fatty Liver Index and ultrasound correlated with worse learning, recall, and concentration. 104 Histologically confirmed steatosis and ballooning were associated with impaired immediate and delayed memory and reduced left hippocampal activity, determined by functional magnetic resonance imaging (fMRI). 105 Concurrent type 2 diabetes shifted the deficit profile toward processing speed and working memory. 106 107 These studies did not assess inflammatory markers, but a cross-sectional study bridges this gap. Elevated CD69 ^+^ CD4 T cells and relative T _h_ 17 enrichment, accompanied by higher CD4-derived IL-17 ex vivo, distinguished MASLD patients with mild cognitive impairment from cognitively intact controls, 108 linking peripheral T cell activation to neuroinflammation and cognitive dysfunction. These studies establish that MASLD impairs cognitive function across multiple domains 109 110 111 ( Table 1 ).

**Table 1:** Human studies on MASLD and neuroinflammation (epidemiology)

Author (y)	Study design; sample size	Neurocognitive tests	Liver assessment	Outcomes	Brain region(s) implicated by data
Seo et al (2016) 104	Cross-sectional No NAFLD *n* = 3,598 46.3% Male NAFLD *n* = 874 53.7% Male	Tests taken from Neurobehavioral Evaluation System 2 by Baker and Letz Simple reaction time test (SRTT) Symbol-digit substitution test (SDST) Serial digit learning test (SDLT)	Gallbladder examination via Toshiba SSA-90A ultrasound with 3.75 and 5.0 MHz transducers NAFLD was determined as moderate-severe steatosis via ultrasound examination in the absence of hepatitis B or C or excessive alcohol consumption (≥1 drink/d for women; 2 drinks/d for men) Liver enzyme activity was assayed by a Hitachi 737 automated multichannel chemistry analyzer (measured in U/L)	NAFLD Independently correlated with worse SDLT scores ALT activity Independently correlated with worse SDLT and SDST scores AST activity Independently correlated with lower SDLT scores	Medial temporal (hippocampus) Prefrontal cortex
Weinstein et al (2018) 106	Cross-sectional No NAFLD *n* = 629 44.2% Male NAFLD *n* = 137 56.9% Male	Brain MRI 1.5-T Siemens Avanto scanner to calculate Brain volume 4 mm contiguous slices • 3D T1 and double echo proton density • T2 coronal Hippocampal volume Covert brain infarcts	CT of fatty liver scoring NAFLD is defined as a liver-to-phantom ratio of 0.33 or less	NAFLD Associated with smaller brain volume: Higher median (25th–75th percentile) CRP: 3.05 (1.38–5.90) mg/L 1.70 (0.81–3.72) mg/L *p* < 0.001	Cerebellum
Weinstein et al (2018) 107	Cross-sectional Controls *n* = 239 42.98% Male NAFLD and T2DM *n* = 174 47.76% Male T2DM only *n* = 142 51.51% Male NAFLD only *n* = 239 50.7% Male	CERAD-WL Word learning subset from the consortium to establish a registry for Alzheimer's disease Assessed immediate and delayed learning ability for new verbal information. Animal fluency test Assessed categorical verbal fluency (component of executive function) Digit symbol substitution test module from Wechsler Adult Intelligence	NAFLD is defined by a fatty liver index (FLI) score ≥ 60 (calculated based on BMI, waist circumference, TAG, GGT levels) in the absence of any other causes of chronic liver disease (e.g., alcoholic liver disease, hepatitis B virus, hepatitis C virus, or iron overload) T2DM is defined as taking diabetes medication or having a fasting glucose measure > 126 mg/dL	NAFLD and T2DM are associated with worse DSST scores: T2DM associated with worse AFT scores: NAFLD without T2DM showed no significant association with cognitive performance levels when compared with a group with neither NAFLD nor T2DM	Dorsolateral prefrontal cortex (DLPFC)
Liu et al (2021) 109	Prospective Over the age of 40, no history of dementia No NAFLD *n* = 856 43.5% Male NAFLD *n* = 795 54.65% Male	Chinese Mini-Mental State Evaluation (MMSE) assessed global cognitive status and cognitive impairment incidence	NAFLD diagnosed by the Asia-Pacific Working Party guideline via Liver ultrasound examination using a B-mode Doppler sonography machine with a 3.5 MHz probe: presence of at least 2 of the following characteristics • Diffusely increased echogenicity of the liver relative to the kidney or spleen • Hepatic vascular blurring • Deep attenuation signs. Absence of other liver diseases or excess alcohol consumption • >20 g/d for men • >10 g/d for women	NAFLD Approximately 1.5-fold risk of developing cognitive impairment over 4 y	NA
Miao et al (2023) 105	Cross-sectional Patients undergoing bariatric surgery Control *n* = 35 20.0% Male MRI data: *n* = 12 Total NAFLD *n* = 190 31.1% Male NAFLD without NASH *n* = 136 32.4% Male MRI data: *n* = 37 NAFLD with NASH *n* = 54 27.8% Male MRI data: *n* = 21	Chinese MMSE Montreal Cognitive Assessment (MoCA), Beijing Version: repeatable battery for the assessment of neuropsychological status (RBANS) Assessed multiple cognitive subdomains (immediate memory, visuospatial constructional, language, attention, delayed memory). Raw scores were age-adjusted. Subdomain scores below 85 were regarded as probable cognitive impairment in that domain. MRI Ingenia 3.0 T CX, Philips Medical Systems, The Netherlands, with an 8-channel head coil. Image processing through MATLAB	Liver histology via biopsy Controls defined by < 5% steatosis and no clinical, biochemical, or imaging evidence of fatty liver disease NAFLD is defined by steatosis in at least 5% of hepatocytes and the absence of excessive alcohol consumption or other chronic liver disease Steatosis scores calculated based on the percentage of hepatocytes with fat droplets • S0 ≤ 5% • S1 = 5–33% • S2 ≥ 33–66% • S3 ≥ 66% Lobular inflammation graded by the number of foci • LI0 = None • LI1 ≤ 2 • LI2 = 2–4 • LI3 ≥ 4 Hepatocyte ballooning scored as • 0 = none • 1 = few balloon cells • 2 = prominent ballooning NAFLD activity score (NAS) is calculated as the sum of steatosis, lobular inflammation, and ballooning scores. NAFLD individuals are categorized by • Non-NASH = NAS < 5 • NASH = NAS ≥ 5	No global cognitive, visuospatial, constructional, language, or attention differences were observed between the control and NAFLD groups. Immediate memory impairment occurrence 63.7%; 37.1%; *p* = 0.003 (all NAFLD vs. control) 63.5%; 34.6% *p* = 0.005; (ballooning vs. no ballooning) 74.1%; 37.1%; *p* = 0.001 (NASH vs. control) OR (95% CI) 2.189 (1.020–4.699); *p* = 0.044 (SH3) 3.655 (1.419–9.414); *p* = 0.007 (ballooning) 4.582 (1.635–12.838); *p* = 0.004 (NASH) Delayed memory impairment occurrence 21.1%; 5.7%; *p* = 0.032 (all NAFLD vs. control) 40.8%; 12.5%; *p* < 0.001 (S3 vs. S0–S2) 31.5%; 5.7%; *p* = 0.004 (NASH vs. control) 31.5%; 16.9%; *p* = 0.026 (NASH vs. NAFLD without NASH) OR (95% CI) 5.498 (2.311–13.081); *p* < 0.001 (SH3) 6.512 (1.229–34.512); *p* = 0.028 (NASH) Brain volumes NASH vs. NAFLD without NASH 3,234.1 ± 275.5 mm ^3^ ; 3,427.7 ± 237.0 mm ^3^ ; *p* = 0.004 (left hippocampus) 289.3 ± 38.9 mm ^3^ ; 313.4 ± 28.1 mm ^3^ ; *p* = 0.013 (left presubiculum) 405.0 ± 31.7 mm ^3^ ; 439.0 ± 38.4 mm ^3^ , *p* = 0.003 (left subiculum) Decreased left hippocampal activation in the context of olfactory stimulation on fMRI in NASH vs. NAFLD without NASH patients	Left hippocampus and subregions
Cushman et al (2023) 110	Prospective No NAFLD *n* = 110 26% Male NAFLD *n* = 213 52% Male	All tests are administered over the phone Six-item screener Used at baseline to determine eligibility Animal fluency test, word list learning test, word list recall test Administered every 2 y during follow-up. Scores were considered impaired if the most recent administration was more than 1.5 standard deviations below the age-, race-, sex-, and education-adjusted mean scores. Incident cognitive impairment is defined as impaired scores on at least two of the three follow-up tests when participants had normal baseline screener results	FLI FLI > 60 in the absence of heavy alcohol intake defines the NAFLD diagnosis FLI < 20 ruled out NAFLD	OR (95% CI) 2.95 (1.05–8.34; cognitive impairment in NAFLD Subjects) 1.86 (0.81–4.25; cognitive impairment in subjects with AST/ALT ratio > 2)	NA
Bao et al (2024) 111	Prospective MAFLD *n* = 155,068 64.0% Male No MAFLD *n* = 248,438 35.1% Male MASLD *n* = 111,938 59.7% Male No MASLD *n* = 291,568 41.0% Male	Diagnosis using ICD-9 and -10 codes Incident • All-cause dementia • Alzheimer's disease • Vascular dementia	FLI Based on BMI, waist circumference, triglycerides, and GGT. A score ≥ 60 indicated hepatic steatosis. MAFLD is defined by hepatic steatosis and at least one of three other criteria, which define corresponding subtypes • Overweight/obese • T2DM • At least two metabolic abnormalities (lean) MASLD is defined by hepatic steatosis along with at least one of five metabolic abnormalities. There is an overlap in the populations diagnosed with MASLD and MAFLD, but they are treated separately for analyses	Vascular dementia risk 1.32 (1.18–1.48; MAFLD) 2.95 (2.52–3.45; MAFLD-Diabetes) 2.01 (1.25–3.22; MAFLD-Lean) 1.24 (1.1–1.39; MASLD) Alzheimer's risk 0.92 (0.84–1.0; MAFLD) 1.46 (1.26–1.69; MAFLD-Diabetes) 0.83 (0.75–0.91; MAFLD-Obesity) All-cause dementia risk 1.8 (1.65–1.96; MAFLD-Diabetes) 0.9 (0.84–0.95; MAFLD-Obesity)	NA

Abbreviations: ALT, alanine aminotransferase; AST, aspartate aminotransferase; FLI, fatty liver index; GFAP, glial fibrillary acidic protein; GGT, gamma-glutamyl transferase; MAFLD, metabolic-associated fatty liver disease; MRI, magnetic resonance imaging; NAFLD, nonalcoholic fatty liver disease; NAS, NAFLD activity score; NASH, nonalcoholic steatohepatitis; PD-1, programmed death-1; T2DM, type 2 diabetes mellitus.

#### Postmortem Studies of CNS T Cell Influx in MASLD

Postmortem analysis provides the most direct neuropathological evidence for CNS T cell infiltration in human MASLD patients. Across two studies examining the cerebellum and hippocampus in the same cohort, meningeal CD4 T cell infiltration tracked steatohepatitis severity in both regions, accompanied by microglial and astrocyte activation, elevated IL-1β and TNF-α, and progressive neuronal loss: Purkinje cell dropout in the cerebellum from steatohepatitis stage (SH1) onward, and caspase-3-mediated apoptosis in hippocampal CA1 neurons from SH2 onward. 112 113 Expression of programmed death 1 (PD-1) on infiltrating CD4 T cells was suggestive of recent TCR engagement. Whether T cells resided within meningeal vessels or dural lymphatics was not resolved, and parenchymal T cell invasion was not demonstrated, leaving the link between meningeal infiltration and neuronal loss inferential. Within these constraints, the data support a model of stage-dependent CD4 T cell infiltration and region-specific neurodegeneration that provides a neuropathological substrate for the cognitive deficits documented epidemiologically and a human correlate for the preclinical mechanisms described in “Hepatosystemic Inflammation Drives Neuroinflammation in MASLD: Evidence from Animal Studies section” ( Table 2 ).

**Table 2:** Postmortem studies illustrating T cell infiltration in the CNS in MASLD patients

Author (y)	Study design; sample size	Brain region examined	Neurological outcomes	Immunological outcomes
Balzano et al (2018) 112	Cross-sectional postmortem Control *n* = 6 100% Male SH1 *n* = 9 88.8% Male SH2 *n* = 6 83.3% Male SH3 *n* = 4 100% Male Cirrhosis without HE *n* = 4 75% Male Cirrhosis with HE *n* = 5 60% Male	Cerebellar cortex layers • Meninges • Molecular • Purkinje neurons • Granular neurons • White matter	Purkinje neuronal density compared with control Control: 100 ± 4% SH1: 84 ± 4%; *p* < 0.05 SH2: 69 ± 3%; *p* < 0.005 SH3: 68 ± 5%; *p* < 0.005 Cirrhosis: 60 ± 2%; *p* < 0.005 HE: 63 ± 3%; *p* < 0.005 Granular neuronal density compared with control Control: 100 ± 2% SH1: 81 ± 2%; *p* < 0.005 SH2: 75 ± 2%; *p* < 0.005 SH3: 79 ± 3%; *p* < 0.005 Cirrhosis: 82 ± 2%; *p* < 0.005 HE: 82 ± 2%; *p* < 0.005	CD4 T cells in cerebellar meninges (cells/mm) compared with controls Control: 1 ± 1 SH1: 10 ± 1; *p* < 0.005 SH2: 12 ± 3; *p* < 0.005 SH3: 9 ± 1; *p* < 0.05 CD4 PD-1+ cells in cerebellar meninges (cells/mm) compared with SH2 patients SH1: 4 ± 1; *p* < 0.05 SH2: 7 ± 1; *p* < 0.005 CD4 CCR6+ cells in cerebellar meninges (cells/mm) compared with SH1 SH1: 3 ± 1 SH2: 7 ± 2; *p* < 0.005 SH3: 4 ± 1; *p* < 0.05 Microglial perimeter (µm) in white matter compared with controls and SH1 Control: 218 ± 7 SH1: 215 ± 5 SH2: 188 ± 5; *p* < 0.05; *p* < 0.001 SH3: 187 ± 5; *p* < 0.05; *p* < 0.001 Cirrhosis: 172 ± 6; *p* < 0.005; *p* < 0.005 HE: 169 ± 5; *p* < 0.005; *p* < 0.005 Microglial perimeter in the molecular layer compared with controls and SH1 Control: 259 ± 12 SH1: 214 ± 6; *p* < 0.005 SH2: 178 ± 6; *p* < 0.005; *p* < 0.005 SH3: 183 ± 8; *p* < 0.005; *p* < 0.05; Cirrhosis: 167 ± 7; *p* < 0.005; *p* < 0.005 HE: 170 ± 9; *p* < 0.005; *p* < 0.001 GFAP area staining (% of control area) in white matter compared with controls Control: 100 ± 2% SH2: 132 ± 2%; *p* < 0.005 SH3: 133 ± 2%; *p* < 0.005 Cirrhosis: 134 ± 3%; *p* < 0.005 HE: 112 ± 4%; *p* < 0.005; *p* < 0.005; *p* < 0.005 (compared with SH2; SH3; Cirrhosis)
Leone et al (2023) 113	Cross-sectional postmortem Control *n* = 6 100% Male SH1 *n* = 9 88.8% Male SH2 *n* = 6 83.3% Male SH3 *n* = 4 100% Male Cirrhosis without HE *n* = 4 75% Male Cirrhosis with HE *n* = 5 60% Male	Hippocampus	Cells stained with NeuN (cells/mm ^2^ ) compared with controls Control: 306 ± 14 SH2: 170 ± 14; *p* < 0.05 SH3: 155 ± 13; *p* < 0.01 Cirrhosis: 168 ± 15; *p* < 0.01 Neurons expressing caspase-3-activated (cells/mm ^2^ ) compared with controls Control: 6 ± 0.9 SH2: 17 ± 1.5; *p* < 0.01 SH3: 14 ± 1.4; *p* < 0.05	Microglial perimeter (µm) in SH2, SH3, and cirrhotic patients compared with controls Control: 272 ± 8 SH2: 167 ± 8; *p* < 0.01 SH3: 198 ± 7; *p* < 0.05 Cirrhosis: 185 ± 9; *p* < 0.05 GFAP area staining (% of total area) compared with controls Control: 24% ± 1% SH1: 42% ± 1%; *p* < 0.0001 SH2: 39% ± 1%; *p* < 0.001 SH3: 49% ± 1%; *p* < 0.0001 Cirrhosis: 43% ± 1%; *p* < 0.0001 IL-1β content (arbitrary units of optical density) in neurons of the CA1 region of the hippocampus compared with controls Control: 0.14 ± 0.01 SH3: 0.21 ± 0.02; *p* < 0.01 Cirrhosis: 0.23 ± 0.01; *p* < 0.01 TNF-α content (arbitrary units of optical density) in neurons of the CA1 region of the hippocampus compared with controls Control: 0.11 ± 0.01 SH3: 0.20 ± 0.01; *p* < 0.05 CD4+ T cells infiltration in hippocampal meninges (cells/mm) compared with controls Control: 2.2 ± 0.3 SH2: 5.4 ± 1.4; *p* < 0.05 SH3: 5 ± 0.6; *p* < 0.05 Monocyte infiltration (cells/mm) compared with controls Control: 6.3 ± 0.4 SH2: 8.0 ± 0.8; *p* < 0.05 SH3: 8.7 ± 0.7; *p* < 0.01 Cirrhosis: 8.3 ± 0.9; *p* < 0.05

Abbreviations: CNS, central nervous system; IL, interleukins; MASLD, metabolic dysfunction-associated steatotic liver disease; TNF-α, tumor necrosis factor-alpha.

### Hepatosystemic Inflammation Drives Neuroinflammation in MASLD: Evidence from Animal Studies

Animal studies have defined several mechanisms by which diet-induced metabolic stress licenses T cell activation in the liver and its draining lymph nodes, and how the resulting inflammatory state propagates to the CNS ( Table 3 ).

**Table 3:** Animal studies supporting the hepatosystemic T cell-neuroinflammation axis in MASLD

ID	Study model (species, diet/intervention, duration)	Axis	Key T cell subset	Main findings
A	Ghazarian et al, 2017; 114 C57BL/6 mice; high-fat diet (HFD); 16 wk. IFNαR1-deficient mice; CD8 ^−/−^ adoptive transfer recipients	Hepatic	Hepatic CD8 T cells licensed by type I IFN signaling	HFD induced a hepatic IFN-I program, expanding CD8 T cells in the liver. CD8-derived IFN-γ and TNF-α impaired hepatocyte insulin signaling. IFNαR1 deficiency reduced steatosis, insulin resistance, and intrahepatic CD8 accumulation. Gut microbial products upstream of hepatic IFN-I are implicated. TCRVβ repertoire analysis supported bystander rather than antigen-driven expansion; T cell origin (local expansion vs. peripheral recruitment) was not resolved.
B	Dudek et al, 2021; 115 C57BL/6 mice; choline-deficient high-fat diet (12 mo) and Western diet confirmatory model	Hepatic	CXCR6 ^+^ PD-1 ^+^ Granzyme B ^+^ resident-like CD8 T cells (formal residency panel not applied)	Identified a metabolically licensed CXCR6 ^+^ CD8 population that mediates TCR-independent hepatocyte killing via Fas/FasL. Delineates the resident CD8 subset converted to cytotoxicity by IL15 and local metabolic milieu
C	Breuer et al, 2020; 116 LDLR ^−/−^ C57BL/6 mice; Western diet (obese/hyperlipidemic NASH model) and lean high-fat choline-deficient C57BL/6 mice; CD8 depletion and adoptive transfer	Hepatic	Hepatic CD8 T cells' pathogenicity depends on the obese metabolic milieu.	In obesity-associated NASH, hepatic CD8 T cells expanded approximately 3.5-fold, acquired a cytotoxic phenotype, and activated hepatic stellate cells (HSCs). CD8 depletion reduced HSC activation and macrophage infiltration; T cell origin not resolved
D	Rai et al, 2020; 117 F11r ^−/−^ (JAM-A knockout) C57BL/6 mice; Western diet; α _4_ β _7_ or MAdCAM-1 monoclonal antibody blockade (mAb); antibiotic perturbation	Hepatosystemic	α _4_ β _7_^+^ CD4 T cells recruited via gut–liver trafficking axis	Western diet expands circulating gut-homing α _4_ β _7_^+^ CD4 T cells and induces microbiota-dependent MAdCAM-1 upregulation in colon and liver. α _4_ β _7_ mAb reduced hepatic CD4 T cell recruitment, improved intestinal barrier integrity, and reduced hepatic inflammation, fibrosis, and metabolic dysfunction. Gut dysbiosis required as a “second hit.” Provides evidence that hepatic T cell accumulation in MASLD is driven by peripheral recruitment
E	Harley et al, 2014; 120 C57BL/6 mice; high-fat diet; IL-17RA knockout and IL-17A neutralization. Segmented filamentous bacteria colonization/depletion	Hepatic	CD4 T _h_ 17/IL17 axis	IL-17 signaling accelerated steatosis to steatohepatitis. IL-17RA deficiency increased steatosis but protected against hepatocellular damage, inflammatory NADPH oxidase induction, and steatohepatitis; IL-17A neutralization was protective. Microbiota inducing IL-17 worsened liver injury, linking gut-driven T _h_ 17 responses to liver injury
F	Gupta et al, 2024; 118 CCl4-induced liver fibrosis model (C57BL/6 mice); α _4_ β _7_ or MAdCAM-1 mAb	Hepatosystemic	α _4_ β _7_^+^ CD4 and CD8 T cells recruited via gut–liver trafficking axis	Extended the α _4_ β _7_ /MAdCAM-1 trafficking beyond diet-induced NASH, showing that fibrotic livers accumulate activated α _4_ β _7_^+^ T cells and that blockade of α _4_ β _7_ or MAdCAM-1 reduces hepatic T cell recruitment, inflammation, and fibrosis progression
G	Kjærgaard et al, 2024; 123 Sprague–Dawley rats high-fat high cholesterol diet (nonfibrotic early MASLD model); 16 wk	Hepatosystemic	Not examined. Systemic cytokine profiling	In nonfibrotic early MASLD, hepatic steatosis with lobular inflammation is accompanied by elevated plasma and CSF CXCL1. Systemic inflammatory signature associated with microglial activation, reduction in prefrontal cortical synaptic density, and impaired recognition memory and depression-like behavior. Shows that prefibrotic hepatic inflammation drives systemic and CNS inflammatory consequences via cytokines
H	Butler et al, 2023; 127 high-fat diet; aged rats (24-mo F344 × BN F1 rats); peripheral CD8 T cell depletion	Systemic/CNS	CD8 T cells	HFD increased brain CD8 T cells in aged rats; peripheral CD8 depletion, without depleting brain CD8 T cells, prevents HFD-induced memory deficits, lowers hippocampal IFNγ and IL-1β, and preserves synaptic proteins. Shows that peripheral CD8 T cells contribute to diet-induced neuroinflammation and cognitive dysfunction, linking systemic metabolic immune activation to CNS injury
I	Ahrendsen et al, 2023; 128 Obese and nonobese human postmortem brains ( *n* = 50); stereotactic AAV-GFP injection into arcuate/ventromedial hypothalamus in FVB mice	CNS	CD8 T cells	Human postmortem data: CD8 T cells accumulate in the hypothalamic median eminence/arcuate nucleus in obesity, especially with diabetes; cytotoxic injury markers are elevated. Mouse data: hypothalamic CD8 T cell recruitment induces rapid weight gain. Identifies the hypothalamus as a CNS target of T cells and supports bidirectional brain-metabolism interactions
J	Becker et al, 2025; 130 C57BL/6 mice; high-fat, high-sugar diet (16 wk); Treg depletion and intracerebral Treg transfer experiments	CNS	FOXP3+ Tregs depleted. CD4 T _h_ 1-like cells expanded.	T _h_ 1/Treg imbalance in the hypothalamus permits diet-induced neuroimmune dysfunction after 8 wk of feeding. Treg reconstitution limited hypothalamic immune activation. Whether local or systemic Treg deficits drove CNS responses was not examined
K	Graindorge et al, 2025; 131 Sprague-Dawley rats; high-fat, high-cholesterol (90 d; early, nonfibrotic MASLD)	CNS	Not examined.	Early MASLD induces neuroinflammation in the cerebellum and frontal cortex, with altered inflammatory mediators, increased microglial markers, and elevated CX3CL1 and Lipocalin-2
L	Poxleitner et al, 2024; 132 wild-type C57BL/6J and APP/PS1 transgenic mice (amyloidosis susceptible); Western diet (6 mo); severe fatty liver	CNS	CD69 ^+^ CD44 ^+^ CD8 T cells in brain parenchyma	Western diet increases activated CD8 T cells in brain parenchyma. CD3 T cells accumulate in the cortex, hippocampus, and hypothalamus, and near the choroid plexus

Abbreviations: CNS, central nervous system; CSF, cerebrospinal fluid; FOXP3, forkhead box P3; IFN, interferon; IL, interleukin; MAdCAM, mucosal addressin cell adhesion molecule-1; MASLD, metabolic dysfunction-associated steatotic liver disease; NASH, nonalcoholic steatohepatitis; PD-1, programmed death-1; TCR, T cell receptor; TNF-α, tumor necrosis factor-alpha.

#### Hepatic T Cell Activation in MASLD

A key question in MASLD immunopathology is whether hepatic T cell accumulation reflects local clonal expansion, peripheral recruitment, or both. Preclinical and clinical studies now provide evidence supporting each of those possibilities. High-fat diet feeding increased hepatic K _i_ -67 ^+^ CD44 ^+^ CD8 T cells within 16 weeks in wild-type mice 114 ( Table 3A ). Gut-derived microbial products were identified as inducers of T cell accumulation via type I IFN signaling. Activated CD8 T cells, in turn, impaired insulin signaling in hepatocytes by producing IFN-γ and TNF-α. TCRVβ repertoire analysis revealed unbiased clonality, suggestive of bystander rather than antigen-driven expansion, placing innate inflammatory signaling upstream of hepatic T cell activation. Corroborating these findings in human MASLD, interferon-related genes, interferon regulatory factor 3 and interferon stimulated gene 15, and intrahepatic CD8 T cell infiltration each correlated with MASLD Activity Score, while CD8 T cell abundance was associated with glycated hemoglobin, linking this innate-adaptive immune axis to metabolic disease severity.

Further evidence for bystander activation of hepatic CD8 T cells in MASLD comes from Dudek et al who demonstrated that the lipotoxic hepatic microenvironment induced a CXCR6 ^+^ PD-1 ^+^ GranzymeB ^+^ CD8 T cell subset in mice fed a choline-deficient high-fat diet or Western diet (WD; Table 3B ). 115 The K _i_ -67 ^−^ and nuclear receptor subfamily 4a1 (Nr4a1)/lymphocyte antigen 6C (Ly6C) ^lo^ phenotype of this subset was consistent with cytokine-mediated activation of tissue-resident memory T cells, though formal residency markers were not applied. IL-15, acetate, and extracellular ATP were identified as upstream activating signals. Neither MHC-I nor PD-1 blockade suppressed CD8-mediated hepatocyte killing, and hepatocytes from β2-microglobulin ^−/−^ mice remained susceptible, establishing TCR-independent and Fas/FasL-dependent cytotoxicity. CXCR6 ^+^ CD8 T cells were enriched in human MASH livers, supporting a conserved role for this subset across mouse and human MASH. 115

Implicating locally activated hepatic CD8 T cells in MASH-driven liver fibrosis, Breuer et al 116 demonstrated that CD8 T cell depletion in WD-fed low-density lipoprotein receptor (LDLR ^−/−^ ) mice reduced hepatic inflammation, hepatic stellate cell (HSC) activation, and macrophage accumulation ( Table 3C ). Sorted hepatic CD8 T cells from MASH mice activated primary HSCs ex vivo, and their adoptive transfer into chow-fed recipients increased α-smooth muscle actin (α-SMA) expression, indicative of a profibrotic role for CD8 T cells. In human MASH, hepatic CD8 T cell density correlated positively with α-SMA across progressive MASH and cirrhosis, extending these findings to human disease. CD8 T cell depletion in a lean choline-deficient mouse model did not decrease hepatic inflammation, fibrosis, or HSC activation, demonstrating that the profibrotic effector function of CD8 T cells is contingent on the hyperlipidemic MASLD milieu rather than a general feature of steatohepatitis.

Parallel to these resident and locally activated programs, active recruitment from the periphery is an independent contributor to hepatic T cell accumulation. Using mice with compromised intestinal epithelial permeability fed a WD that develop more advanced steatohepatitis within a short period of feeding, Rai et al showed that WD increased circulating α _4_ β _7_^+^ CD4 T cells, which were recruited to both intestine and liver through microbiota-dependent upregulation of mucosal addressin cell adhesion molecule-1 (MAdCAM-1; Table 3D ). 117 Diet-induced dysbiosis of the mucosa-associated microbiota, marked by Proteobacteria expansion, correlated with elevated MAdCAM-1 expression in colonic mucosa and liver, and antibiotic treatment reduced MAdCAM-1 expression and hepatic inflammation and fibrosis, linking microbial composition to T cell trafficking. Monoclonal antibody blockade of α _4_ β _7_ decreased CD4 T cell recruitment to gut and liver, improved intestinal barrier integrity, and attenuated steatohepatitis, fibrosis, and metabolic dysfunction. MAdCAM-1 blockade produced similar effects. α _4_ β _7_^+^ CD4 and CD8 T cells also accumulated in CCl4-induced fibrotic liver, and blockade of either α _4_ β _7_ or MAdCAM-1 reduced T cell recruitment and attenuated fibrosis ( Table 3F ). 118 MAdCAM-1 blockade also reduced hepatic monocyte influx, an effect attributable to MAdCAM-1 binding to L-selectin via its mucin-like domain, indicating immunoregulatory control of myeloid cell trafficking. 119 In human MASH liver, MAdCAM-1 expression correlated with integrin subunit alpha 4/beta 7 (genes encoding α4β7) transcripts, identifying this axis as a therapeutic target.

The collective evidence supports a model in which hepatic T cell pathology in MASLD reflects two converging processes: bystander activation through cytokine-mediated reactivation of tissue-resident populations, and microbiota-integrin-dependent peripheral recruitment ( Fig. 3 ). Superimposed on this is a state of immune dysregulation in which T _h_ 1 and T _h_ 17 expansion shifts the hepatic cytokine environment toward IFN-γ and IL-17A production 125 ( Table 3E ), while concurrent impairment of FOXP3 ^+^ Tregs, the central tolerance regulators described in “Education in the Thymus section,” erodes immune restraint. 121 122 Peripheral recruitment and local activation thus operate not as independent inputs but within a dysregulated intrahepatic T cell compartment that amplifies both. Whether these mechanisms are differentially weighted at distinct disease stages, and whether their relative dominance determines fibrotic trajectory or responsiveness to therapy, are questions the current literature does not resolve.

**Fig. 3:**
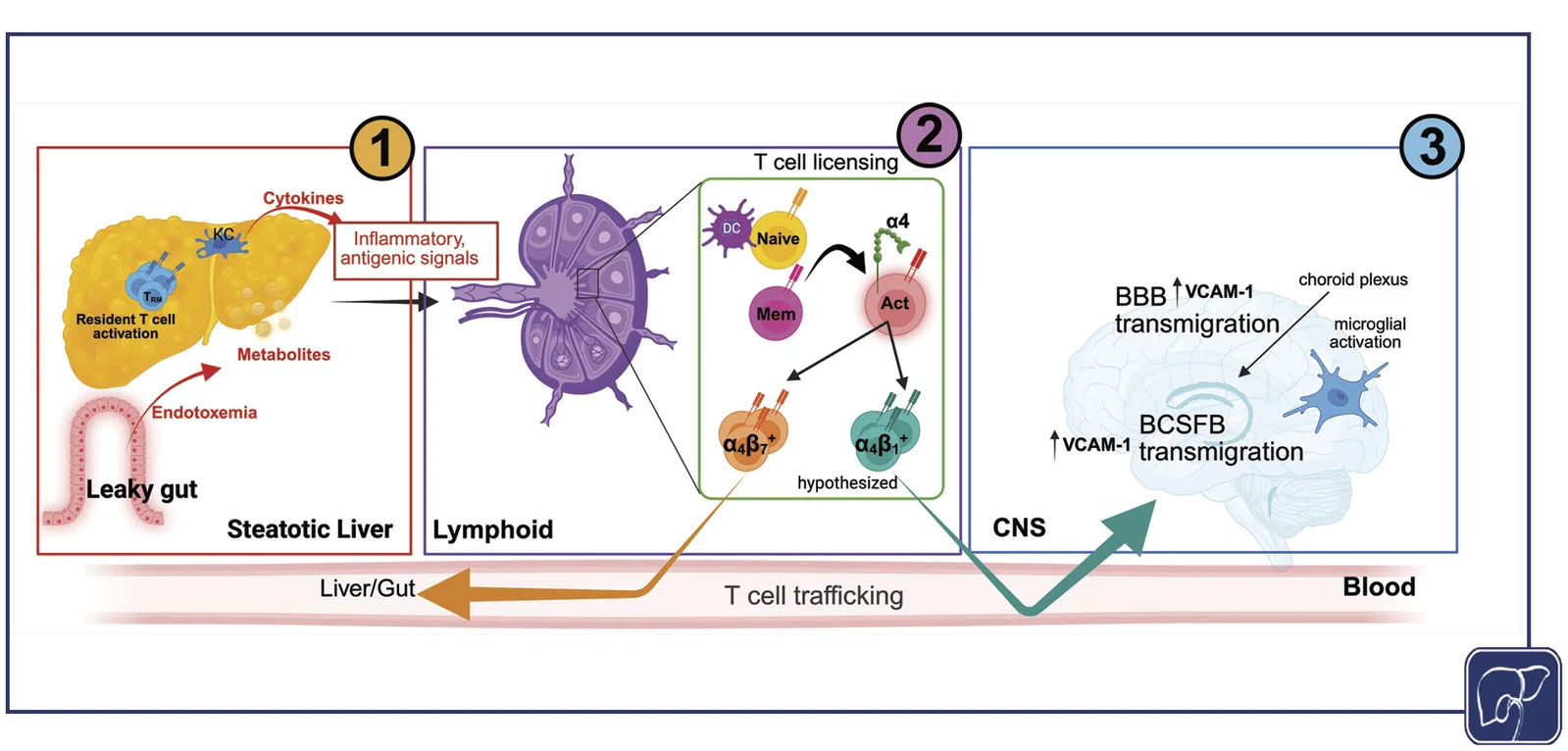
T cell activation and CNS entry routes in MASLD. Gut dysbiosis promotes lipopolysaccharide translocation into portal circulation (endotoxemia), activating hepatic Kupffer cells (KC) and triggering release of cytokines and metabolites within the steatotic liver. Resident memory T cells (T _RM_ ) are activated in situ (Panel 1). Inflammatory and antigenic signals from the liver reach draining lymphoid tissue, where dendritic cell (DC)-mediated antigen presentation licenses naive T cells, producing activated (Act) and circulating memory (Cir Mem) subsets that upregulate the α4 integrin subunit (Panel 2). T cells that pair α4 with β7 (α _4_ β _7_^+^ ) are recruited back to the liver and gut; those that pair α4 with β1 (α _4_ β _1_^+^ ) are directed toward the CNS. Systemically elevated cytokines upregulate VCAM-1 at CNS barriers, permitting α _4_ β _1_^+^ T cell entry via blood–brain barrier (BBB) transmigration and blood–CSF barrier (BCSFB) transmigration into the CSF (Panel 3). Infiltrating T cells drive microglial activation, amplifying neuroinflammation. Source: *Created in BioRender. Iyer, S. (2026)* *https://BioRender.com/10n2z65*

#### Hepatic-to-Systemic Inflammatory Propagation in MASLD

How the intrahepatic inflammatory state propagates beyond the liver is the question to which we now turn. In a prefibrotic MASLD model, hepatic steatosis with lobular inflammation alone was sufficient to increase CSF CXCL1 levels, activate microglia, reduce synaptic density, and impair cognition, indicating that early hepatic push signals in MASLD precede structural CNS pathology ( Table 3G ). 123 Hepatic lipotoxicity activates TLR4/NF-κB/MyD88 signaling, driving KC-derived TNF-α, IL-6, and IL-1β into the blood, resulting in activation of CNS barriers. 124 NOD-like receptor protein 3 inflammasome-derived IL-1β, generated in both hepatic and visceral adipose tissue, constitutes yet another proximal driver, engaging microglial IL-1R1 to induce hippocampal neuroinflammation and impair long-term potentiation. 125

Circulating cytokines, chemokines, and hepatokines then converge on the CNS through immune activation and barrier activation, propagating the hepatosystemic inflammatory state into the brain parenchyma. 126 Cytokines, however, are not the only systemic effectors. Peripheral T cells constitute a discrete cellular arm of this hepatosystemic-to-CNS propagation. Peripheral CD8 T cell depletion prevents high-fat diet-induced brain CD8 T cell accumulation in the presence of an intact BBB and rescues hippocampal- and amygdala-dependent memory deficits in rats, indicating that CNS T cell influx in MASLD reflects active immune surveillance rather than passive barrier breakdown ( Table 3H ). 127

#### Neuroinflammation: Hypothalamus and Beyond

Where do peripheral T cells go once licensed for CNS entry? The circumventricular organs with constitutively permeable BBB are exposed, and the hypothalamus exemplifies this vulnerability ( Table 3I ). 128 129 Postmortem examination of brains from obese individuals revealed CD8 T cell accumulation in the median eminence and arcuate nucleus, with the highest burden in those with concurrent diabetes. This infiltration was shaped partly by progressive loss of local immunoregulation: a decrease in hypothalamic FOXP3 ^+^ Tregs was detectable after as few as 8 weeks of high-fat, high-sugar diet, preceding overt obesity ( Table 3J ). 130 In tandem, hypothalamic CD4 T cells shifted toward a T _h_ 1 state, while microglia and infiltrating monocyte-derived macrophages upregulated MHCII, CD80/86, and CD40, hallmarks of ongoing inflammation. That Treg reconstitution limits hypothalamic immune activation and reverses diet-induced metabolic impairments positions hypothalamic Treg loss upstream of neuroinflammatory progression.

Hypothalamic vulnerability is not an isolated finding. In rats fed a high-fat, high-cholesterol diet for 90 days, induction of steatosis, hepatocyte ballooning, and lobular inflammation without fibrosis resulted in the modulation of 36 inflammatory mediators in the cerebellum and 17 in the frontal cortex, with microglial expansion in both regions ( Table 3K ). 131 Induction of CX3CL1/Fractalkine and Lipocalin-2 (Lcn2) indicated that early MASLD induced a neuroinflammatory microenvironment primed for T cell recruitment. Parenchymal T cell infiltration followed as a downstream consequence of this progressive barrier disruption. In wild-type and amyloid precursor protein/presenilin1 (APP/PS1) transgenic mice fed a WD for 6 months, CD69 ^+^ CD44 ^+^ CD8 T cells accumulated in brain tissue, and CD3 immunohistochemistry showed infiltration in the cortex, hippocampus, hypothalamus, and choroid plexus ( Table 3L ). 132 This distribution is consistent with choroid plexus remodeling driven by systemic inflammation and with evidence that Lcn2, elevated in MASH, disrupts choroid plexus barrier integrity. 133

These studies support the following hypothesis: MASLD generates a hepatic and visceral inflammatory milieu from which effector T cells and cytokines enter the systemic circulation ( Fig. 4A ). In early disease, choroid plexus remodeling and upregulation of endothelial adhesion machinery render CNS barriers progressively permissive to peripherally licensed lymphocytes ( Fig. 4B ). Sustained microglial activation and glia limitans remodeling then enable parenchymal infiltration, with T cell accumulation in regions mirroring human postmortem findings ( Fig. 4C ). What no study has yet established is whether hepatically primed T cells can be traced into specific brain lesions. The need for this cellular continuity is addressed in the next section.

**Fig. 4:**
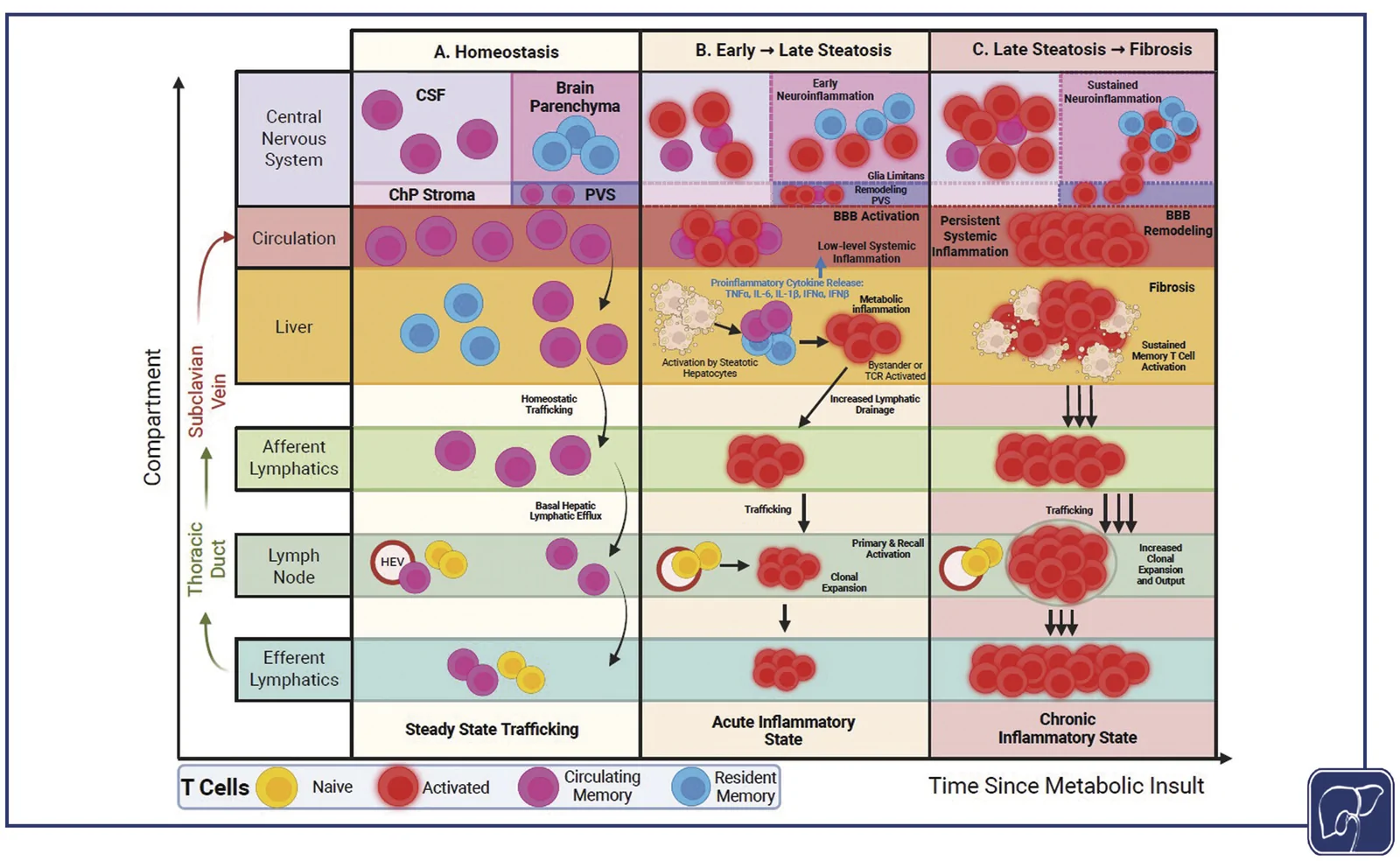
Temporal evolution of T cell trafficking across the liver–brain axis in MASLD. T cell dynamics are mapped across six anatomical compartments against three disease stages: homeostasis, early-to-late steatosis, and late steatosis-to-fibrosis. Under ( **A** ) homeostasis, hepatic resident memory T cells undergo basal lymphatic egress via the thoracic duct and subclavian vein; naive T cells traffic through draining lymph nodes via HEV, and circulating memory T cells constitutively patrol CNS border compartments without parenchymal entry. During early-to-late steatosis ( **B** ), metabolic inflammation (TNF-α, IL-6, IL-1β, IFN-α/β) activates hepatic memory T cells through bystander or TCR-dependent mechanisms, increasing lymphatic output and driving clonal expansion in draining lymph nodes primed by gut-derived antigens. The resulting activated T cell pool mediates BBB endothelial activation and early neuroinflammation, with glia limitans remodeling at the PVS. In late steatosis and fibrosis ( **C** ), sustained memory T cell reactivation and amplified clonal expansion generate persistent systemic inflammation, BBB remodeling, and sustained parenchymal neuroinflammation. T cell color coding: yellow, naive; red, activated; pink, circulating memory; blue, resident memory. BBB, blood–brain barrier; ChP, choroid plexus; CSF, cerebrospinal fluid; HEV, high endothelial venule; PVS, perivascular space. Source: *Created in BioRender. Iyer, S. (2026)* *https://BioRender.com/h3qi4qb*

### Therapeutic Implications and Future Directions

MASLD increases the risk for neuroinflammation and cognitive decline, with T cell licensing of CNS access as a key purported mechanism within the liver-to-brain inflammatory axis. Several questions warrant answering before this biology can be translated into therapeutic targets ( Fig. 5 ).

**Fig. 5:**
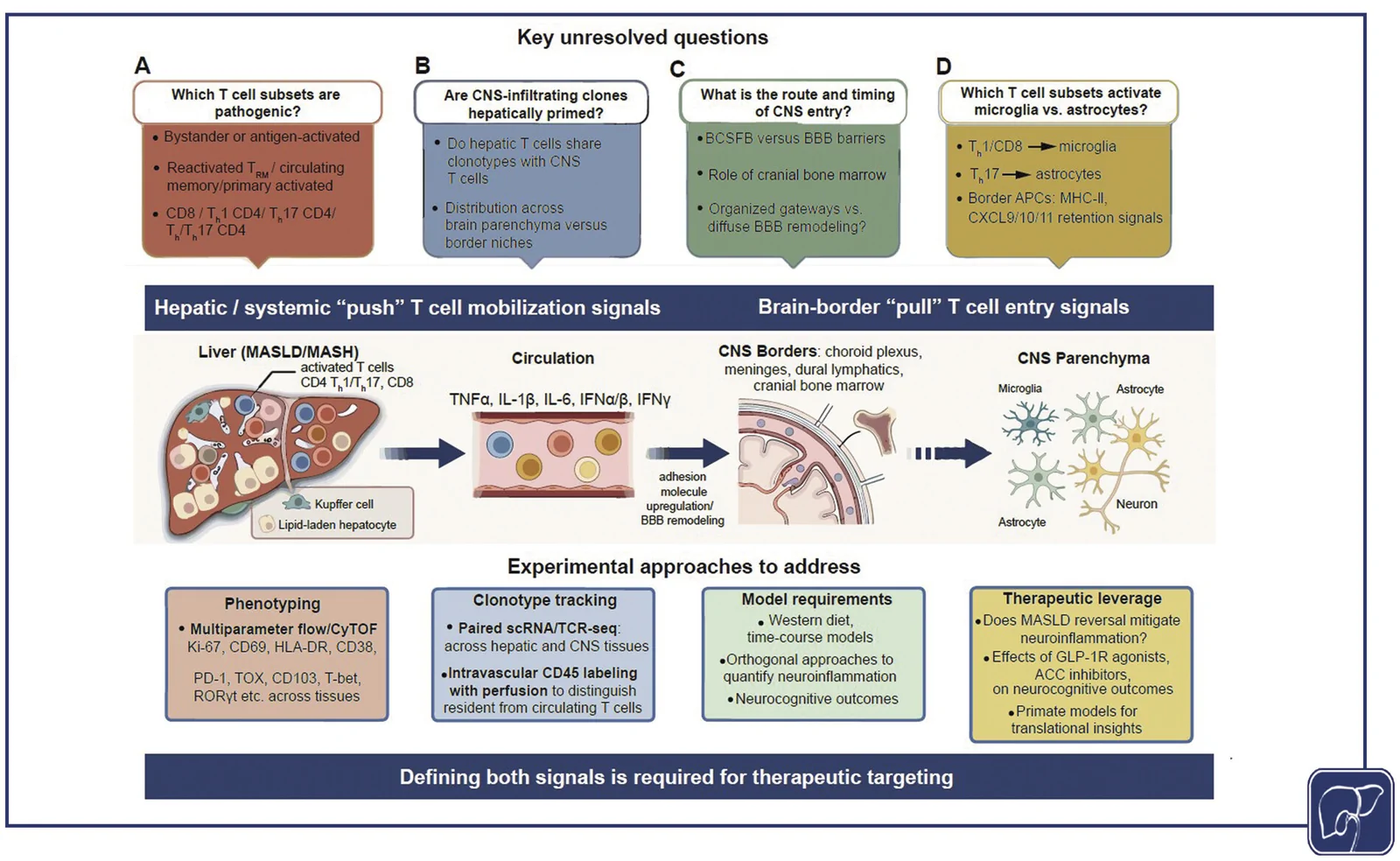
Key unresolved questions and experimental priorities in MASLD-associated neuroinflammation. The figure is organized in three tiers. The top tier identifies four mechanistic gaps: the identity and activation state of pathogenic T cell subsets, whether CNS-infiltrating clones are hepatically primed, the route and timing of CNS entry, and which T cell subsets drive microglial versus astrocyte activation. The middle tier depicts the push-pull axis through which hepatic and systemic inflammatory signals (TNF-α, IL-1β, IL-6, IFN-γ) mobilize activated CD4 T _h_ 1/T _h_ 17 and CD8 T cells from the liver into circulation, where adhesion molecule upregulation and BBB remodeling at CNS border compartments (choroid plexus, meninges, dural lymphatics, cranial bone marrow) license parenchymal entry and T cell-glial interactions. The bottom tier outlines experimental approaches to resolve these questions: multiparameter flow cytometry/cytometry by time-of-flight (CyTOF)-based T cell phenotyping across tissues, paired scRNA/TCR-seq for clonotype tracking, intravascular CD45 labeling with perfusion for compartmental discrimination, physiologically relevant dietary models with neurocognitive endpoints, and assessment of hepatic therapeutic interventions on neuroinflammatory outcomes. Defining both the hepatic push and brain-border pull signals is required for rational therapeutic targeting. ACC, acetyl-CoA carboxylase; APC, antigen-presenting cell; BBB, blood–brain barrier; BCSFB, blood–CSF barrier; GLP-1R, glucagon-like peptide-1 receptor; HFD, high-fat diet; LN, lymph node; T _RM_ , tissue-resident memory T cell.

Which T cell populations drive CNS pathology in MASLD, and what is their functional state ( Fig. 5A )? Distinguishing bystander cytokine-activated cells (CD69 ^+^ , CD25 ^hi^ , PD-1 ^−^ ) from antigen-activated cells (CD69 ^+^ , CD25 ^hi^ , PD-1 ^+^ , HLA-DR ^+^ , CD38 ^hi^ ) is necessary to identify the source of T cell activation. 134 135 K _i_ -67 and CD69 mark activated T cells broadly, and PD-1 expression without thymocyte selection-associated high mobility group box protein co-expression does not distinguish antigen activation from early exhaustion. 136 137 Tissue-resident populations (T _RM_ : CD69 ^+^ , Kruppel-like factor 2 ^low^ , CD62L ^−^ , CCR7 ^−^ , with CD103 [α _E_ β _7_ ]) must be distinguished from circulating T cells to localize where activation occurs. 78 138 139 Among CD4 T cells, T follicular helper designation requires CXCR5 and B cell lymphoma-6, not PD-1 alone, 140 141 and T-bet ^+^ T _h_ 1, RORγt ^+^ T _h_ 17, and T _h_ 1/T _h_ 17 subsets must be resolved given their distinct effector programs and roles in neuroinflammation. 74 142 Whether CNS-infiltrating T cells share clonotypic identity with those expanding in the liver is a key unanswered question; paired single-cell RNA/TCR-seq across liver, blood, meninges, and brain would resolve this ( Fig. 5B ). Intravascular CD45 labeling with time-course flow cytometry across liver, draining lymph nodes, gut-associated lymphoid tissue, cervical lymph nodes, choroid plexus, meninges, and brain parenchyma will provide the spatial and temporal resolution currently lacking. 143

When do T cells gain CNS access in MASLD, by which route do they enter, and how do they instruct glial cells once there? The choroid plexus, meninges, cranial bone marrow, and dural lymphatics each represent distinct immunological gateways with unique APCs, barrier properties, and chemokine gradients for T cell recruitment and retention. 144 Which gateway predominates, and at what disease stage, is unknown ( Fig. 5C ). Paired plasma and CSF sampling in longitudinal MASLD cohorts, stratified by fibrosis and inflammation stage, would provide clinical correlates of CNS barrier involvement and T cell trafficking. Neuroinflammation imaging, including translocator protein positron emission tomography as a microglial activation readout, would add spatial resolution to these biomarker data. In addition, the interaction of T cells with glial cells has not been examined in MASLD. IFN-γ from T _h_ 1 cells drives microglial NF-κB activation, while microglia and border-associated myeloid cells reciprocally present antigen via MHC-II and release CXCL9/10/11, reinforcing T cell retention. 108 112 113 145 T _h_ 17 cytokines activate astrocytes, which secondarily amplify microglial recruitment. 146 Which T cell subsets drive each arm of this response, and whether they can be selectively targeted, are important questions to address ( Fig. 5D ).

Can the neuroinflammatory and neurocognitive changes initiated by MASLD be reversed? Dietary reversal of hepatic steatosis and inflammation is documented, 147 but whether this resolves CNS immune infiltration, microglial activation, and synaptic injury remains unknown. The question becomes sharper in the context of pharmacological intervention. Do interventions for metabolic diseases such as acetyl-CoA carboxylase (ACC) inhibitors, 148 FXR agonists, 149 GLP-1 receptor agonists, 150 thyroid hormone receptor-β agonists, 151 FGF19, 152 and anti-fibrotic agents in clinical development, 153 confer neuroprotective benefit through modulation of the liver–brain immune axis, or does CNS pathology persist once established? Answering this requires longitudinal cohorts that couple liver histology or validated noninvasive fibrosis assessment with serial cognitive testing and peripheral T cell phenotyping. Ongoing liver-directed intervention trials should incorporate cognitive batteries and neuroimaging endpoints to determine whether hepatic disease modification translates to CNS benefit—a question the current trial designs are not built to answer.

## Conclusion

The evidence reviewed here supports the model advanced in the abstract: T cell licensing by hepatosystemic signals represents the immune arm of the liver–brain axis and a mechanistic bridge between MASLD and neuroimmune dysfunction. Hepatic inflammation generates a systemic cytokine milieu that conditions T cells in the hepatic draining lymph nodes, reprogramming their integrin and chemokine receptor expression and metabolic state to enable CNS access. The neuroinflammatory pull signals at CNS border compartments complete the circuit. Human postmortem data confirm the downstream consequence: meningeal CD4 T cell infiltration, microglial activation, and region-specific neurodegeneration tracking steatohepatitis severity. While cellular continuity between hepatically licensed T cells and specific CNS lesions remains to be established, the combined evidence across epidemiological, neuropathological, and preclinical studies is sufficient to define a mechanistic framework and act on it therapeutically.

That framework positions therapeutic targets at three nodes, with upstream intervention as the most tractable near-term priority. Liver-directed agents—FXR agonists, GLP-1 receptor agonists, ACC inhibitors, and thyroid hormone receptor-β agonists, several of which are approved or in late-stage development for MASH—are the most immediately actionable. Reducing hepatic inflammation upstream is also the mechanistically cleanest intervention: if licensing depends on hepatic cytokine output and draining lymph node conditioning, attenuating that signal should constrain the entire cascade. Midstream blockade of integrin-dependent trafficking at the α4β7/MAdCAM-1 and α4β1/VCAM-1 axes offers a cellular checkpoint with proven precedent in inflammatory disease, but context-dependence of these pathways, as demonstrated by the paradoxical effects of α4 blockade in SIV infection, 85 demands careful mechanistic validation before clinical translation in MASLD. Downstream CNS-directed strategies targeting microglial activation and barrier adhesion machinery remain the least defined tier and are likely to be most relevant once hepatic inflammation has already established a neuroinflammatory state. The central translational question this model makes answerable is whether reversing the hepatic source is sufficient, and if so, at what disease stage intervention must occur to prevent irreversible CNS pathology.

As an aging population carries an expanding burden of metabolic disease, the window for intervention, before neuroinflammation consolidates into irreversible neurodegenerative change, is closing. This demands that the field move from cataloguing associations to defining the cellular mechanisms that can be targeted and to testing whether reversing hepatic inflammation is sufficient to halt the CNS pathology it initiates.

## Notes

Lay Summary People with chronic metabolic diseases are at increased risk for brain aging and cognitive decline. Understanding the mechanisms underlying this progression is important so that we can develop therapies to slow neurodegeneration. There is ample evidence from other diseases that inflammation within the central nervous system, also known as neuroinflammation, accelerates brain aging. This review synthesizes what is known in the liver disease literature about immune activation, focusing on a subset of adaptive immune cells called T cells. We make the case that inflammation originating in the liver spreads through the body, a process we term hepatosystemic inflammation, and that this systemic inflammatory state, mediated by T cells, may contribute to neuroinflammation and cognitive decline in metabolic dysfunction-associated steatotic liver disease.
